# Cognitive Behavioral Therapy Improves Physical Function and Fatigue in Mild and Moderate Chronic Fatigue Syndrome: A Consecutive Randomized Controlled Trial of Standard and Short Interventions

**DOI:** 10.3389/fpsyt.2021.580924

**Published:** 2021-04-12

**Authors:** Merethe Eide Gotaas, Tore C. Stiles, Johan Håkon Bjørngaard, Petter C. Borchgrevink, Egil A. Fors

**Affiliations:** ^1^Department of Circulation and Medical Imaging, Faculty of Medicine and Health Sciences, Norwegian University of Science and Technology, Trondheim, Norway; ^2^National Competence Centre for Complex Symptom Disorders, St. Olav's University Hospital, Trondheim, Norway; ^3^Department of Psychology, Norwegian University of Science and Technology (NTNU), Trondheim, Norway; ^4^Department of Public Health and Nursing, Faculty of Medicine and Health Sciences, Norwegian University of Science and Technology, Trondheim, Norway; ^5^Faculty of Nursing and Health Sciences, Nord University, Levanger, Norway

**Keywords:** CFS, chronic fatigue syndrome, CBT, fatigue, physical function, myalgic encephalitis

## Abstract

**Objective:** To study whether standard cognitive behavioral therapy (CBT) and a shorter, interpersonal oriented cognitive behavioral therapy (I-CBT) can improve physical function and fatigue in patients diagnosed with mild to moderate chronic fatigue syndrome (CFS) in a multidisciplinary fatigue clinic.

**Design:** Consecutively 236 participants 18–62 years old meeting the Centre of Decease Control, CDC 1994 criteria, with a subsample also fulfilling the Canadian criteria for CFS, were randomly allocated to one of three groups. Two intervention groups received either 16 weeks of standard CBT or 8 weeks of I-CBT vs. a waiting-list control group (WLC). Primary outcome was the subscale Physical Function (PF) from SF-36 (0–100). Secondary outcome was amongst others fatigue measured by Chalder Fatigue Questionnaire (CFQ) (0–33). Outcomes were repeatedly measured up to 52 weeks from baseline.

**Results:** The additional effect relative to baseline at post-intervention for SF-36 physical function was 14.2 (95% CI 7.9–20.4 *p* < 0.001) points higher for standard CBT and 6.8 (0.5–13.2 *p* = 0.036) points higher for I-CBT compared with the control group. The additional effect relative to baseline at post-intervention for fatigue was 5.9 (95% CI 0.5–10.5 *p* = 0.03) points lower for standard CBT compared with the control group but did not differ substantially for I-CBT 4.8 (95% CI −0.4 to 9.9 *p* = 0.07). The positive change in physical function persisted at 1-year follow-up for both treatment groups, and for standard CBT also in fatigue. The two intervention groups did not differ significantly in self-reported physical function and fatigue at the 1-year follow-up. No serious adverse reactions were recorded in any of the groups during the trial period.

**Interpretation:** A 16-week standard, individual CBT intervention improves physical function and fatigue in CFS outpatients with mild to moderate disease. A shorter 8-week I-CBT program improves physical function. Both treatments are safe, and the effect persist 1 year after baseline.

**Clinical Trial registration:** ClinicalTrials.gov, Identifier: NCT00920777, registered June 15, 2009.

REK-project number: 4.2008.2586, registered April 2, 2008. Funding: The Liaison Committee for Education, Research and Innovation in Central Norway.

## Introduction

Chronic fatigue syndrome/myalgic encephalomyelitis (CFS/ME), is an illness characterized by unexplained, severe fatigue and post exertional malaise with additional symptoms including, cognitive impairment, sleep disturbance, sensory hypersensitivity, headache, pain in muscles and joints, irritable bowel and intermittent flu-like symptoms (1–4). The illness severely impairs the patients‘ daily functioning both socially and in terms of income acquisition (5).

The terms CFS and ME are often used interchangeably and in combination, CFS/ME, ME/CFS. Until recently, there has been no international consensus on whether ME and CFS are separate conditions or similar enough to belong under an umbrella term such as CFS/ME (6). In this study CFS means all these terms, according to the new ICD-11 classification (7). Many different criteria sets are used to diagnose CFS, such as the Oxford and Sharpe 1991, CDC 1994, Reeves 2003, Canadian Consensus Criteria, NICE 2007, the International Consensus Criteria 2011 (8) and the ‘systemic exertion intolerance disease‘, SEID criteria 2015 (2, 3). There is no scientific basis to claim that some criteria are more accurate than others to classify a CFS phenotype (9, 10). In ICD-10 the code G 93.3 “post viral fatigue syndrome” and “benign myalgic encephalomyelitis” is used for the illness, but no criteria are specified (11). The newly published ICD-11 also adds the term chronic fatigue syndrome (CFS) to these and codes it 8E49 with the parent “other disorders of the nervous system” (7).

Due to terminological variations and diagnostic inconsistencies, it is difficult to assess the prevalence and incidence rate of CFS in a population. Overall prevalence estimates worldwide vary from 0.1 to 2.5%, depending on the diagnostic criteria used (1, 12, 13).

The etiology of CFS is largely unknown. Nevertheless, several possible predictors and associative mechanisms have been hypothesized, including various infections, prolonged strain and physical trauma (14, 15), an autoimmune etiology (16), as well as leaky gut and microbiome (17). Several molecular neuro-biological approaches have also been made to explore the etiology of CFS (18). As of today, there is no known biomarker to diagnose CFS, and a biopsychosocial model has been taken into consideration for both understanding, treatment and rehabilitation (19). Clinical examination and blood tests are directed toward confirming or excluding other possible clinical conditions.

According to systematic reviews, *individual* cognitive behavior therapy (CBT) and graded exercise therapy (GET) have until now been the best-documented treatments for mild to moderate CFS, also in combination, but the effect has been found to be moderate (20–23). However, some of these studies have been debated and criticized, due to methodological issues such as patient selection, inclusion criteria and lack of objective outcome measures (24). Group CBT therapy has also shown moderate improvement in CFS-patients (25). However, due to few randomized studies with varying quality and the use of different sets of criteria for diagnosing CFS, there is no consensus whether CBT is an effective and safe treatment for all patients with CFS (20, 26–28). On the other hand, few adverse effects are found in CBT treatment. In a reanalysis of three RCTs on CBT for CFS, it was concluded that patients receiving CBT did not experience more frequent or more severe symptom deterioration than untreated patients and that the reported deterioration during CBT seemed to reflect the natural variation in symptoms (27). In spite of that, a small review by Twisk et al. have suggested worsening in patients with CFS after CBT and GET (29). Thus, the reporting of non-serious adverse events may reflect the nature of the illness rather than the effect of treatments (30).

This study aims to increase knowledge on whether standard and interpersonal oriented individual cognitive behavioral therapy can be an effective treatment for mild and moderate chronic fatigue syndrome, and whether a presumptive positive outcome persists in 1 year from baseline. To investigate this question, we designed a consecutive randomized controlled trial with a 1-year follow-up to compare a standard CBT and a shorter, interpersonal CBT (I-CBT) vs. a waiting list control group (WLC) and assess the possible treatment effect sustainability. Systematic reviews on treatment and management of chronic fatigue syndrome have found little evidence concerning the appropriate duration of follow-up of interventions used in the management of CFS (31, 32). However, as CFS is a long-term condition it would be sensible to follow up participants for an appropriate period. The relapsing nature of CFS suggests that follow-up should continue for at least 6–12 months after the intervention period has ended. It is of importance to confirm that the improvement observed has been due to the intervention itself and not just to a naturally occurring fluctuation in the course of the illness. There are few RCT studies with CBT for CFS with 1-year follow-up assessments and especially few if any, with individual CBT and waiting list controls (32–35). The inclusion criteria, number of patients and length, content and administration of therapy vary a great deal between the trials. This has made it a challenge to compare and generalize the findings. We have not found that any of the existing meta-analysis on CBT for CFS have examined treatment length as an important variable. In a Cochrane systematic review from 2008, four studies offered eight or fewer sessions of CBT, and two studies offered more than eight sessions of CBT (36). A highly significant difference in effect of similar magnitude was shown for both CBT groups when compared with usual care. We hypothesized that both standard CBT and I-CBT would have statistically significant effects on physical function as the primary outcome, as well as secondary outcomes e.g., fatigue, compared with the WLC group in CFS diagnosed with the Centre of Decease Control, CDC 1994 and Canada criteria (12, 37). We also hypothesized that there would be no statistically significant difference in primary and secondary outcomes between standard CBT and I-CBT. If it turns out to be no significant difference in outcome after the I-CBT and standard CBT interventions, the shorter therapy can be a less strenuous and more cost-effective treatment, as has been studied in other psychotherapy studies (38). The prospect of a less weary treatment may be of special importance to patients with CFS and may help make this type of intervention more accessible. We aimed to assess the effect and safety of both treatments.

## Materials and Methods

### Study Design and Participants

This study was a consecutive randomized controlled open-label trial with outcome assessed up to 52 weeks after baseline for patients meeting the CDC 1994 criteria (37) for CFS.

**Figure 1:**
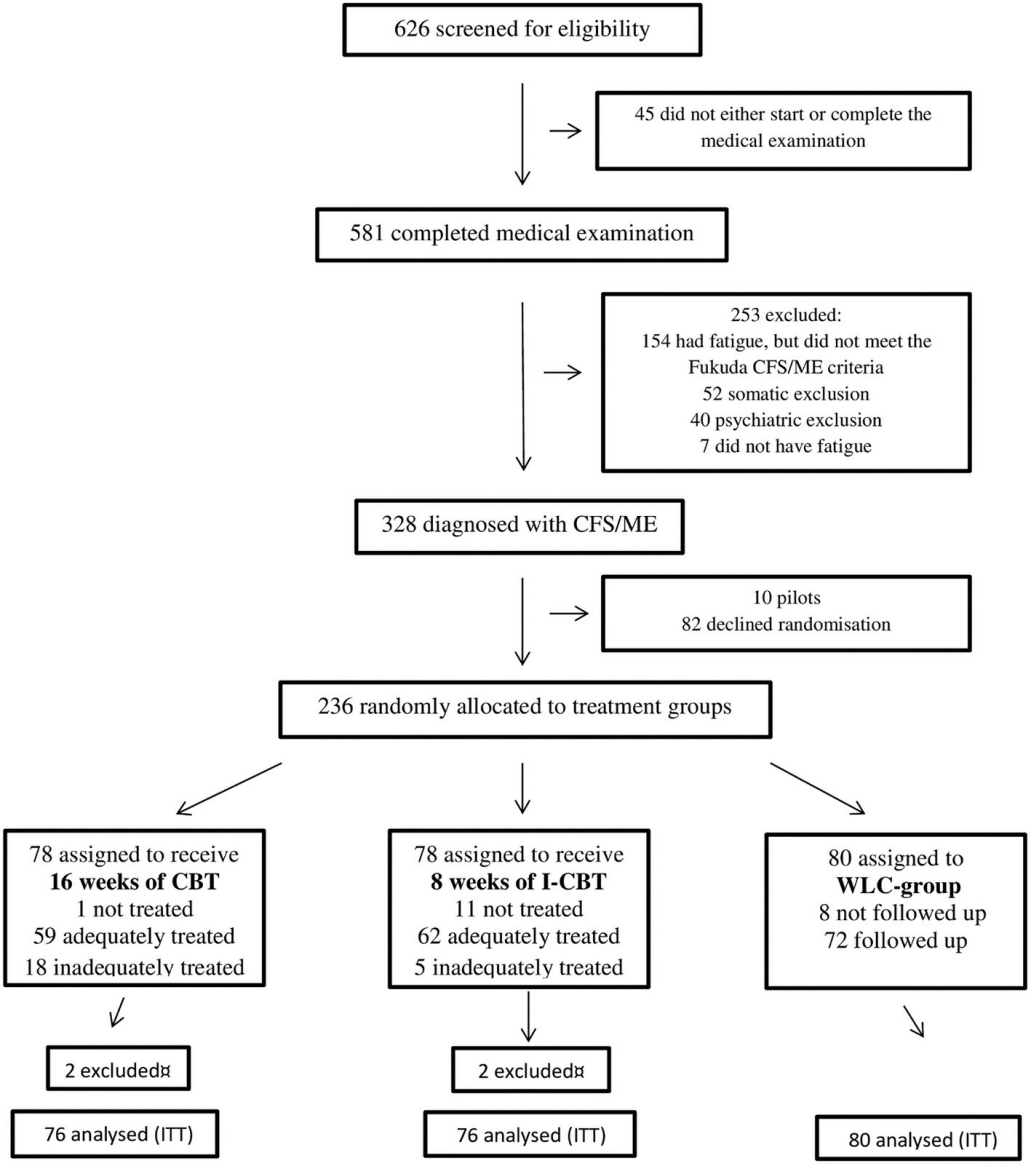
Consort flow chart.

The trial was registered and approved by the Norwegian Regional Committee for Medical and Health Research Ethics (REC Central/NTNU) April 2, 2008, before inclusion of patients. There was a delay in registration of the trial in Clinical Trials (June 15, 2009), because the research group was not aware of the registration requirements at the time. The authors confirm that all ongoing and related trials for this intervention are registered. In all, 626 patients with unspecified fatigue referred from their GP were assessed for eligibility at the CFS/ME clinic at St. Olav‘s Hospital in Trondheim between October 6, 2008, and September 12, 2012 (Figure 1). Of the 328 assessed patients who met the CDC 1994 criteria for CFS, 236 were recruited and gave their consent to be included in the trial (10 were included in a pilot study and 82 declined randomization). In the last year of inclusion, participants were also assessed by the Canada 2003 criteria (39). A total of 110 of 132 patients (83.3%) diagnosed in this period were found to meet both the CDC 1994 and Canada 2003 criteria for CFS. Of these 110 patients, 74 gave their consent to be included in the trial, while 36 declined randomization. Outcome data collection was completed on September 26, 2013.

### Procedure

All referrals to the clinic were screened by an experienced multidisciplinary team consisting of a doctor (general practitioner), a clinical psychologist or psychiatrist, a physical therapist, and a registered nurse. Blood samples were taken from each patient in accordance with the Norwegian Directorate of Health's requirement for assessing patients with suspected CFS (40), including numerous immunology, serology, and standard clinical chemical test (See Appendix S5). The tests were primarily done for the purpose of identifying patients with CFS, but some of the test results will also be utilized in later articles generated from this study to answer relevant research questions.

The physical therapist tested the patient's physical condition, including an indirect VO2max test, muscle tonus, tender points and a brief neurological examination. The psychologist or psychiatrist used a structured interview M.I.N.I (International neuropsychiatric interview) (41) to assess and evaluate the patients' mental health status in addition to a clinical psychological examination. The physician‘s main task was to detect specific medical and psychiatric disorders and, in such cases, refer them to other medical or psychological treatments (37). Based on the multidisciplinary examination, the team thereafter decided whether the patient met the CDC 1994 criteria for CFS, and later also the Canada 2003 Criteria, and thus did not have any other medical or psychological conditions which explained their CFS symptoms, including disabling fatigue and post-exertional malaise (PEM). Patients who did not meet the CDC 1994 criteria or were pregnant, younger than 18 years or older than 62 years were excluded. Patients who were unable to attend hospital outpatient appointments or did not speak fluent Norwegian were also excluded.

### Randomization and Masking

After baseline assessment and having obtained a written consent, participants were allocated to either one of the two treatment groups or to the control group by computer-generated sequence, provided by the Section for Applied Clinical Research (ACR) at NTNU. Once notified of treatment allocation by the ACR, the research coordinator informed the participant, first by phone and later in a letter including practical information and a treatment plan. The participants started treatment within 3 weeks after randomization. Participants and therapists could not be masked to treatment allocation. Both therapist and participant knew the length and contents of the therapy that was given. The participants scored the primary and secondary outcomes in questionnaires. The statistician was masked to treatment groups when analyzing the outcome data, but the difference in number of measuring points made total masking a challenge.

### Interventions

Cognitive behavioral therapy (CBT) is a form of psychotherapy with assumed moderate effect for many patients with mild to moderate CFS (20). Standard CBT, also called “classical,” “Beckian,” or “second wave” CBT seeks to reduce and eliminate the symptoms of problems by changing behaviors associated with “automatic thoughts,” emotions and physical symptoms (e.g., pain or fatigue) (42). The cognitive behavioral model hypothesizes that it is not a situation in and of itself that determines what people feel and perceive, but rather the way in which they construe a situation which may influence the physical symptoms (43, 44).

The 16-week intervention of individual, standard CBT was given by three trained cognitive therapists at St. Olav's Hospital, Trondheim, Norway. The actual therapy manual was based on previous manuals from similar trials (35, 45), and associated with the fear avoidance (FA) theory of CFS (46). The FA theory focuses on the link between cognitive and behavioral responses. The intervention consisted of 16 weekly sessions plus a booster session 4 weeks after the 16th session. The following main elements were included in the intervention: (a) information and explanation of the CFS symptoms based on contemporary knowledge on both physical and psychological components, (b) agreement on goals for the treatment (c) reading of self-help guides about how to cope with chronic fatigue (d) avoidance of excessive activity or rest and/or sudden change in activity, (e) planning of regular, predictable, continuous and graded activity to prohibit deconditioning, (f) recovering self-confidence and self-control by starting with a 5-min walk morning and night followed by gradual development (GET), and (g) standard CBT-procedures (47). Graded activity is used analogs with “graded exercise therapy” (GET) in this study, as the activity program was not primarily traditional sports exercises, but rather gradual activity exercises anchored to the fear avoidance model (48).

The 8 weeks of individual, interpersonal and personality-oriented CBT was given by four trained cognitive therapists at the private health centre Coperio, Trondheim, Norway. The treatment manual was developed by co-author (TCS). The intervention consisted of 8 weekly therapy sessions and a booster session 4 weeks after the eighth session. I-CBT is a new form of treatment for CFS/ME. Its treatment effects have thus not been tested before. Since standard CBT has been shown to have limited effects in previous outcome studies, it was considered to compare standard CBT with a more interpersonal oriented approach. Although the focus of I-CBT is on more interpersonal issues, it is still the traditional CBT approach of identifying how they think, feel and behave in the situation. Moreover, underlying beliefs are identified and challenged. The treatment was based on a biopsychosocial model (49) in which elements from interpersonal cognitive therapy (50) and personality-guided therapy (51) were integrated. It assumes that treatment must be individualized to match the prominent personality style of the patient. Four personality styles were identified: Cautious, achievement oriented, identity or role confused and disease oriented. The overarching goal of the treatment was to give the patient insight into own personal goals and needs, to know their true inner self, and how to improve their interpersonal relatedness toward others, including family, friends, work relations and health and welfare authorities. The following elements were included in the intervention: (a) information and explanation of the CFS symptoms based on contemporary knowledge on both physical and psychological components with specific emphasis on the goals and needs of the self, its ability to emotion regulate and to actively improve relationships with others, (b) agreement on long-term goals related to self-satisfaction and interpersonal functioning rather than immediate symptom reduction and increase in physical function, and (c) focus on individualized personality-guided and interpersonally oriented factors that were assumed to create painful and difficult experiences to the individual.

The control group was a waiting-list group in which the participants waited for 16 weeks after baseline before they were post-scored and then offered 8 weeks of I-CBT outside the study. The ideal would have been a control period up to 1 year without intervention, but this was not possible due to ethical considerations.

Supervision/internal validity CBT was delivered by clinical psychologists and one psychiatrist. Supervision in both I-CBT (TCS) and standard CBT (EAF) was given to the therapists as a group once a month, or individually if required. All treatment sessions for standard CBT were audio-recorded to make quality assurance and CBT guidance possible. Adequate use of standard CBT was quality assured by an independent observer using the CTACS quality assuring instrument (52). All treatment sessions for I-CBT were recorded on tape to make quality assurance by the head professional advisor possible. The therapists in both treatment groups registered the number of therapy sessions attended for each patient, active withdrawals from treatment, and dropouts from follow-up. Specialist medical care physicians from the CFS outpatient clinic were available to the patients if needed throughout the study period.

### Assessments

The primary outcome measure in this study was the SF-36 version 2 physical function subscale, one of eight subscales (53), presented as a so called “mean difference, additional effect relative to baseline at post- intervention.” In SF-36, raw score data are converted to a scale range of 0–100, where the highest score reflects best self-rated function. An important secondary outcome was global fatigue measured by the Chalder fatigue questionnaire (54). In the Chalder fatigue questionnaire, there is a Likert scoring 0, 1, 2, 3 on 11 items; range 0–33, where the highest score reflects high fatigue. Both the primary and secondary outcome measures are valid and reliable and have been used in numerous trials worldwide, including CFS studies (21, 36, 54). Other secondary outcomes than fatigue were the remaining SF-36 measures/subscales of bodily pain; role physical and general health, vitality, social functioning, role emotional and mental health. The first three subscales reflect the patient‘s physical health, while the last four reflect the mental health. All measures were self-rated by the participants. The objective tests, such as an indirect VO2max test, muscle tonus, muscular tender points and a brief neurological examination were obtained for clinical use and later articles.

### 16-Week CBT

Assessments were made at baseline (0 weeks), just after randomization, at mid-therapy (13 weeks), post therapy (20 weeks/post-intervention), 4 weeks after booster (29 weeks), and 1 year after baseline (52 weeks).

### Eight-Weeks I-CBT

Assessments were made at baseline (0 weeks), just after randomization, post therapy (12 weeks), 4 weeks after booster (21 weeks/post-intervention), and 1 year after baseline (52 weeks).

### Waiting-List Control Group

Assessments were made at baseline (0 weeks), just after randomization, halfway through the waiting period (10 weeks), and at the end of the 16 weeks waiting period (18 weeks/post-intervention).

#### Safety Monitoring: Adverse Reactions and Adverse Events (Harms)

For safety monitoring, we registered adverse events, adverse reaction to trial treatment, and active withdrawal from treatment. Adverse events and adverse reactions were defined as any negative clinical symptom change, disorder, or disease occurring during the study period whether related to the treatment or not. The participant-rated Clinical Global Impression scale was used to assess change in overall health during the trial period (Clinical Global Impressions Scale (CGI-ECDEU, 1976; CIPS, 1986) (55). The therapists also recorded events, if any, in the participants' journal. The therapists also reported to the study coordinator and the medical director of the study. In need of further medical assessment, the patients were instructed to contact their GP or local hospital.

### Statistical Analysis

We calculated sample size assuming a mean change in SF-36 physical function subscale score of 10, 5, and 0 from baseline of, respectively, short I-CBT, standard CBT, and WLC group, a ‘within cell‘ standard deviation (SD) on 15 and a correlation between baseline and post-intervention on 0.3 (*r*-squared on 0.1). For a two-sided test with 5% significance level and 80% power, we calculated that we needed 40 patients in each group, 120 in total. Assuming a maximum drop-out level of 25%, we calculated that we needed an additional number of 18 patients in each group, 174 patients in total. A mean change in SF-36 physical function score of 10, 10, and 0 for standard, short and WLC groups, respectively, would increase power to 90%.

A clinically or minimal important difference (MID) was defined as an improvement of >10 points for the “mean difference, additional effect relative to baseline at post-intervention” or a mean score in SF-36 physical function of >75 post-intervention (56, 57). Norwegian reference values for physical function in SF-36 exists (58), but are calculated with a different edition of SF-36 (version 1) than the one used in our study (i.e. version 2). As an example of a similar population to compare with, the SF-36 physical function version 2 score for the UK working age population is 84, with a SD of 24. Thus, a SF-36 score equal to or above the mean minus 1 SD will be considered in the normal range (score of 60 or more) (56). That would make a SF-36 physical function score of 75 or more within normal range (56).

For the Chalder fatigue scale, a MID was not pre-defined, but 0.5 of the SD of these measures at baseline is described in literature (57, 59). In our study, this equates to 3 points for Chalder fatigue scale (rounded up from 2.64). Normal range for fatigue was defined as less than the mean plus 1 SD scores of the general Norwegian population of 12.2 (+3.9) (60). That is equivalent to a fatigue score of 16.1 or less.

All patients who were enrolled and randomly allocated to treatment were included in the analysis and analyzed in the groups to which they were randomized (intention to treat/ITT) except the patients excluded for becoming pregnant during the trial (n = 4). Due to repeated measures of outcomes, we used a multilevel linear regression model with random slopes in STATA 11 for Windows (Stata Corp., College Station, TX). This method uses all available information during follow-up and is less susceptible to bias from missing responses under the assumption of missing at random. Each follow-up time-point was added to the model as a dummy variable (i.e., 10–13 weeks, 18–21 weeks, 52 weeks and the baseline as a reference). To assess differences between intervention groups and the waiting-list group during follow-up, interaction terms between the group allocation and each registration time-point were included in the model. We also tested a model with only participants with complete registrations of the outcome on all follow-up time points (i.e., “per protocol,” not presented in figures or tables). To assess outcome clustering at the patient level, we estimated an intraclass correlation coefficient (ICC) (61).

### Role of the Funding Source

The funding source, The Liaison Committee for Education, Research and Innovation in Central Norway, had no role in the study design, data collection, data analysis, data interpretation, or writing of this report/article. All the named authors had access to the anonymised data and commented on drafts during the process leading to the final report. The research group had the final responsibility for the decision to submit the report for publication.

### Ethics

The study is approved by the Norwegian Regional Committee for Medical and Health Research Ethics (REC Central/NTNU) in Norway, no 4.2008.2586. All patients gave written consent to participate in the study. Patients randomized to the control group were offered 8 weeks of individual I-CBT after the predefined waiting period of 16 weeks was completed. The ideal would be to have a control period up to 1 year after baseline without intervention, but this was not possible due to ethical considerations.

## Results

Of the 626 patients assessed for eligibility, 581 (93%) completed medical and psychological examination, while 45 (7%) patients did not start or complete the assessment. Thereafter 253 (40%) patients were excluded for the following various reasons: Patients with fatigue, but who did not meet the CDC 1994 criteria for CFS numbered 154 (24%). Respectively, 52 (8%) and 40 (6%) patients were excluded because of somatic and psychiatric disorder. Seven patients did not have fatigue and where thus mis-referred. That left 328 (52%) assessed patients who met the CDC 1994 criteria for CFS, and of these 236 (72%) were recruited to the study (Figure 1). One-way analysis of variance between groups (ANOVA) show that patients' characteristics at baseline were balanced between the intervention groups (physical function SF-36 *p* = 0.919, age *p* = 0.055, fatigue *p* = 0.718, HADS depression *p* = 0.474, HADS anxiety *p* = 0.811 and duration of illness *p* = 0.087). There were no statistically significant differences at baseline between dropouts and non-dropouts (physical function SF-36 *p* = 0.527, age *p* = 0.210, fatigue *p* = 0.861, HADS depression p =0.750, HADS anxiety *p* = 0.656, and duration of illness *p* = 0.086).

Differences for categorical variables, tested with Chi-squares show that there was a higher percentage of women in the standard group than the I-CBT group *p* < 0.05, but no significant difference in years of education *p* > 0.05 (Table 1). All participants identified racially as white. In the last year of inclusion, the patients were classified according to both the CDC 1994 and Canada 2003 criteria for CFS, and 110 of 132 patients (83.3%) were found to meet both the CDC 1994 and Canada 2003 criteria. Of these 110 patients, 74 were included in the trial, 26 declined randomization. There were no statistically significant differences in baseline characteristics between the patients who met both sets of criteria compared to those who were classified only according to the CDC 1994 criteria (physical function SF-36 *p* = 0.236, fatigue *p* = 0.705, HADS depression *p* = 0.202 and HADS anxiety *p* = 0.891). There were neither no statistically significant differences in physical function SF-36 *p* = 0.291 scores, HADS depression *p* = 0.254, HADS anxiety *p* = 0.254 or fatigue *p* = 0.521 scores when comparing the groups later at 20 weeks from baseline. Regardless, the reported anxiety and depression symptom levels were low, and neither of the groups scored a mean level of anxiety or depression over a cut-off point of >8/21 in HADS at baseline (62).

**Table 1:** Demographic and clinical characteristics of the participants at baseline.

	**I-CBT 8**	**CBT16**	**WLC**
	**(*n =* 76)**	**(*n =* 76)**	**(*n =* 78)**
Mean age (SD)	37 (11)	37(11)	32 (10)
Number female n (%)^a^	53 (70)	69 (91)	67 (84)
Education bachelor or higher n (%)	24 (32)	28 (37)	20 (25)
Mean duration of illness in years (range)	4.5 (0.5–14)	5.3 (0.8–13)	4.6 (0.5–13)
Mean HADS depression score (SD)	6.6 (4.1)	5.9 (3.6)	6.6 (3.8)
Mean HADS anxiety score (SD)	6.7 (3.4)	6.8 (3.6)	7.1 (4.1)

*HADS-A and HADS-D, Hospital Anxiety and Depression Scale (range 0–21, where 21 is maximum)*.

a *Differences n (%) for categorical variables, tested with Chi-squares: Number female, I-CBT vs. standard CBT P < 0.05. There were no other statistically significant differences between the groups*.

### Dropouts

Twenty (8.5%) participants dropped out just after randomization and had only registered baseline data. Thereafter, 21 (9%) participants dropped out during treatment. There was a variation in drop-out frequency between the study groups. In the 8-week I-CBT group, five of 78 (6%) patients dropped out during treatment due to fatigue (*n* = 1), lack of motivation for the treatment (*n* = 2), and long distance to treatment centre (*n* = 2). In the 16-week CBT group, 16 of 78 (20.5%) participants dropped out during treatment due to fatigue (*n* = 3), poor economy (*n* = 2), psychosocial strain (*n* = 2), long distance to treatment centre (*n* = 2), lack of motivation for the treatment (*n* = 4), change of therapist (*n* = 1), current infection (*n* = 1), and need of further psychiatric evaluation (*n* = 1). Poor economy in this context means that the participants could not afford to pay for transport to the clinic and therefore could not attend therapy sessions. All baseline data and outcomes registered were analyzed for all three groups.

### Primary Outcome

**Figure 2:**
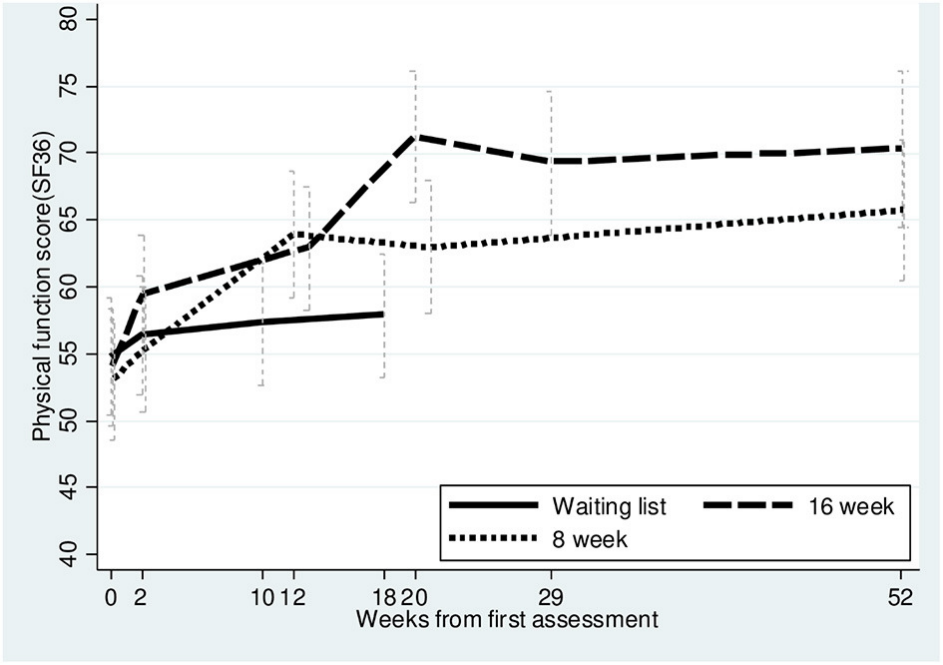
Primary outcome, physical function SF-36 up to 52 weeks.

Standard CBT participants reported a statistically significant increase in their physical function compared to the WL control group, and reached a mean additional effect relative to baseline at post-intervention of 14.2 points, which exceeded the predefined MID improvement number of >10 (57). Correspondingly, the I-CBT reached a statistically significant additional effect relative to baseline at post-intervention of 6.8 points compared to the WLC-group. At 1-year follow-up, paired *t*-tests show that both standard CBT [diff. 0.4 *p* = 0.35 (95%CI −1.5 to 2.3)] and I-CBT [diff. 3.2 *p* = 0.056 (95% CI −0.1 to 6.5)] groups revealed an unchanged, positive effect on physical function from post-intervention. Between-groups analyses regarding physical function found no significant differences between the treatment groups (95% CI −10.1 to 2.1 *p* = 0.20) at the 1-year follow-up (Table 2 and Figure 2).

**Table 2:** Primary outcome data: physical function score (SF-36).

	**I-CBT 8**	**CBT 16**	**WLC**
**Physical function score SF-36**			
Baseline	53.1 (48.5–57.6)	54.0 (49.6–58.4)	54.8 (50.5–59.1)
Post-intervention^*^	62.9 (58.0–67.9)	71.2 (66.3–76.1)	57.9 (53.2–62.5)
Follow-up 1 year	65.7 (60.5–71.0)	70.3 (64.5–76.1)	
Mean difference, additional effect relative to baseline at post-intervention	6.8 (0.5–13.2) *p* = 0.036	14.2 (7.9–20.4) *p* < 0.001	

*Table data show mean scores at three timepoints in addition to mean difference, additional effect relative to baseline post-intervention with a 95% CI. SF-36 (0–100), higher score; fewer symptoms, better function*.

* *Post-intervention: (I-CBT 8: 21 weeks from baseline. CBT 16: 20 weeks from baseline. WLC 18 weeks from baseline). Follow-up: (52 weeks from baseline)*.

### Secondary Outcomes

#### Chalder Fatigue Questionnaire

**Figure 3:**
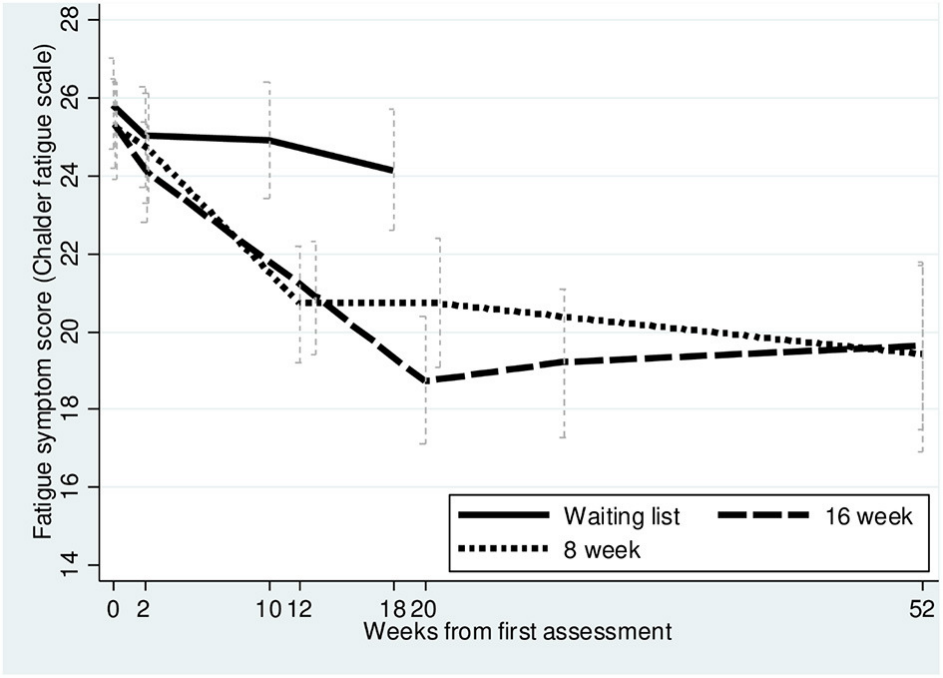
Secondary outcome, Fatigue (CFQ) up to 52 weeks.

Participants in both treatment groups reported less fatigue than the control group and exceeded the defined MID on 3.0 points reduction in fatigue post-intervention 20 weeks from baseline (post-intervention). The mean additional effect relative to baseline for fatigue at post- intervention was 5.9 (statistically significant) and 4.8 (not statistically significant) points lower compared to WLC for standard CBT and I-CBT, respectively (Table 3 and Figure 3). I-CBT did not differ substantially from WLC post-intervention (95% CI −0.4 to 9.9 *p* = 0.07), however, the between-groups analyses showed no significant mean score differences for fatigue at the 1-year follow-up (95% CI −2.8 to 2.1 *p* = 0.8). Eventually, paired *t*-tests showed that both standard CBT [diff. 0.4 (95% CI −1.5 to 2.3 *p* = 0.68)] and I-CBT [diff. −1.4 (95% CI −3.0 to 0.2 *p* = 0.0823)] revealed a sustained reduction in fatigue from post-intervention to 1 year follow up.

**Table 3:** Secondary outcome data: fatigue score (CFQ).

	**I-CBT 8**	**CBT 16**	**WLC**
**Fatigue score**			
Baseline	25.2 (23.9–26.4)	25.3 (24.2–26.5)	25.8 (24.7–27.0)
Post-intervention^*^	20.7 (19.1–22.4)	18.7 (17.1–20.4)	24.1 (22.6–25.7)
Follow-up 1 year	19.3 (17.4–21.2)	19.7 (17.4–21.9)	
Mean difference, additional effect relative to baseline at post-intervention	4.8 (−0.4 to 9.9) *p =* 0.07	5.9 (0.5–10.5) *p =* 0.03	

*Data are mean scores and mean difference, additional effect relative to baseline post intervention with a 95% CI. Chalder Fatigue Questionnaire (0–33), lower score, fewer symptoms, better function*.

* *Post-intervention: (I-CBT 8: 21 weeks from baseline. CBT 16: 20 weeks from baseline. WLC 18 weeks from baseline). Follow-up: (52 weeks from baseline)*.

#### Compared to Norm Scores

At post-intervention, 50% of the standard CBT group and 40% of the I-CBT group reached a predefined SF-36 physical function score of 75 points or more at post-intervention, compared to only 20% in the WLC group (57). Furthermore, 31% of the patients in the standard CBT group, 19% of the patients in the I-CBT group and 10% of the patients in the WLC- group reported normal fatigue scores related to the Norwegian population at the end of the waiting period (a fatigue score of 16.1 or less) (60).

##### SF-36 Sub-dimensions

For the standard CBT group, there were statistically significant positive changes in five of the seven remaining subdimensions for the SF-36 post-intervention scores (i.e., mental health, bodily pain, role physical, social functioning and vitality) compared to the control group. The most profound improvements were in the “role physical,” “social function,” and “vitality” subdimensions. There were no statistically significant changes in “general health and role emotional” compared to WLC. Eventually, there were no statistically significant changes in any of the remaining seven SF-36 subdimensions for the I-CBT group compared to the control group (Table 4).

**Table 4:** Secondary outcome data: SF-36 subscales.

	**I-CBT 8**	**CBT 16**
Mental health	3.6 (−1.9 to 9.2)	**6.9** (1.3–12.3)
General health	6.5 (−0.1 to 13.0)	5.2 (−1.5 to 11.4)
Role emotional	−3.5 (−14.1 to 7.1)	2.1 (−9.9 to 11.1)
Bodily pain	1.8 (−5.6 to 9.1)	**8.6** (1.4–15.8)
Role physical	7.2 (−2.3 to 15.6)	**16.5** (7.6–25.4)
Social functioning	6.3 (−4.3 to 16.8)	**22.7** (12.0–32.8)
Vitality	6.1 (−0.8 to 13.0)	**13.1** (6.3–19.3)

*Table data show mean difference, additional effect relative to baseline post-intervention with a 95% CI. SF-36 (0–100), higher score; fewer symptoms, better function. (I-CBT 8: 21 weeks from baseline. CBT 16: 20 weeks from baseline. WLC 18 weeks from baseline). Statistically significant findings are highlighted*.

### Clinical Global Impression Scale and Adverse Events

More patients rated themselves as “much better” or “very much better” in overall health measured by the Clinical Global Impression Scale (CGI) after both the standard CBT and I-CBT interventions compared to the control group. At post-intervention, 33 and 26% in the standard CBT vs. I-CBT group, respectively, rated themselves as “much better” or “very much better” in overall health compared to 8% in the WLC group. A minority (<5% in each group) rated themselves as “worse” or “very much worse,” but this did not differ substantially between the three groups at post-intervention (Table 5). The registered adverse events were increased fatigue and worsening of other existing CFS such as nausea, headache and pain in muscles and joints.

**Table 5:** Participant-rated clinical global impression of change in overall health (CGI).

	**I-CBT 8**	**CBT 16**	**WLC**
Post-intervention^*^			
Positive change	15 (26%)	17 (33%)	5 (8%)
Minimum change	40 (71%)	34 (65%)	54 (87%)
Negative change	2 (3%)	1 (2%)	3 (5%)
**1-year follow-up**			
Positive change	17 (30%)	11 (23%)	
Minimum change	37 (65%)	32 (67%)	
Negative change	3 (5%)	5 (10%)	
Odds ratio (positive change vs. negative or minimum change post score¤) compared with WLC	4.1 (1.4–12.1) *p* = 0.011	5.5 (1.9–16.3) *p* = 0.002	

*Data are n (%) or odds ratio (95% CI). Positive change was defined as “very much better” or “much better.” Minimum change was defined as “a little better,” “no change,” or “a little worse.” Negative change was defined as “much worse” or “very much worse.”*

* *Comparisons made at “post-intervention”: (I-CBT 8: 21 weeks from baseline. CBT 16: 20 weeks from baseline. WLC: 18 weeks from baseline)*.

## Discussion

Participants who received either 16 weeks of standard CBT with graded activity/exercise or 8 weeks of interpersonal CBT reported improved physical function post-intervention compared to a waiting list control group. The standard CBT participants also perceived less fatigue post- treatment. The positive effect of these statistically significant findings sustained for 1 year. The WLC group was not scored at 1-year follow-up. We discussed the option to have outcomes measured at 12 months also for the WLC group, but decided not to because they all received 8 weeks of I-CBT immediately after the end of the waiting period due to ethical considerations The standard CBT improved most of the secondary outcomes in contrast to I-CBT and WLC. Our findings are consistent with other CBT-treatment trials involving patients diagnosed with mild to moderate CFS, which have shown clinical important improvements in both self-reported physical function and fatigue post-intervention (20, 21). For the standard CBT intervention, the reintroduction to activity was graded and systematic, which also implied a tailored plan of escalation based on each patient's individual level of physical function measured at baseline. An individualized plan with focus on graded, systematic escalation of activity may explain some of the positive effect for the physical function. Our findings are supported by previous studies which have suggested that both CBT and GET can improve physical function and fatigue in some patients diagnosed with mild to moderate CFS (26). In I-CBT the goal was to resume a normal level of activity *without* a graded or systematic approach. Despite the different approach to activity, a general focus on being active in both treatment groups could explain the positive and sustained effect in physical function for both groups at the 1-year follow-up. Forty-eight percent of the participants had a SF-36 physical function mean score of 75 or more at 1-year follow-up. For fatigue, 35% of the participants were in normal range at 1-year follow-up compared to reference values for the Norwegian population (58).

Both treatments were well-tolerated. According to the CGI measures, very few rated themselves as ‘worse or ‘very much worse‘ post-intervention (*n* = 1–2, i.e. 2–3%). Furthermore, their experiences did not vary between the groups including the WLC group. In contrast, 33% in the standard CBT group, 26% in the I-CBT group and only 8% in the control group reported to be “much better or very much better” post-intervention. At the 1-year follow-up, as many as 23% in the standard CBT group and 30% in the I-CBT group rated themselves as “much better or very much better” in overall health.

The effectiveness of cognitive behavioral therapy does not imply that CFS is psychological in nature. Before inclusion the patients were assessed by a psychologist or psychiatrist to rule out any psychiatric disorder as a cause of fatigue and other symptoms. The clinical examination and the HADS-scores showed that symptoms of anxiety and depression were generally low in these patients throughout the trial period. Based on this knowledge, one could assume that the SF-36 PF and fatigue outcome effects were not caused by changes in psychological variables.

The differences in effect on physical function and the other SF-36 subdimensions between the groups, could be interpreted as a positive tendency in favor of standard CBT, even though they were not evident in between group analysis at 1-year follow up. The effect differences between the CBT and I-CBT interventions at post-intervention could be explained by a time dosage-response effect, alternatively as a consequence of different contents in the two therapy modes. At post-intervention, the standard CBT group had received 16 sessions of therapy, while the I-CBT group had got nine, including one booster session. Leveling of the results over time to a non-significant difference in both primary and secondary outcomes between the treatment groups at 1-year follow-up, indicate that CBT has a sustained positive effect on physical function and fatigue in these patients in general, regardless of differences in length and content of therapy. The higher dropout rate in the standard CBT group compared to the I-CBT group (20.5 vs. 6%) may also be due to either duration of, or differences in content of therapy. However, in this trial, regardless of cause, the patients show a lower dropout rate for I-CBT than standard CBT, which may indicate a better compliance in I-CBT. However, since we did not measure compliance explicit in this study, it is just a hypothesis. Nevertheless, analyses showed no statistically significant differences in baseline characteristics when comparing dropouts to non-dropouts. No serious adverse events or reactions were recorded in any of the groups. This suggests that CBT, if delivered as described in the manuals by similarly qualified and trained clinicians, is a well-tolerated and safe treatment for patients with mild to moderate CFS. The finding that many of our participants reported “minimal change” at post-intervention and at the 1-year follow-up suggests that treatment effects can differ between subgroups. It would be of interest to explore whether special characteristics can be found in patients who benefit from CBT, and thus indicate who should be referred to therapy with a prospect of improvement. This is also thermalized in other studies (63).

Our findings were strengthened by the study design (randomized, controlled trial, RCT) the relatively high number of participants, the standardized multi-disciplinary assessment by trained physicians, psychologists and physical therapists before inclusion, the use of manual-defined CBT provided by competent clinicians, the thorough blood-test examinations as well as the use of CTACS as a quality-assuring instrument.

Our trial also had some limitations: There was a delay in registration in Clinical Trials, because the research group was not aware of the registration requirements at the time. The inclusion of patients started 5 months after registration and approval from the Regional Ethics Committee. We excluded patients who were unable to attend the hospital out-patient clinic, which meant that only out-patients with mild to moderate CFS were included. We ended up with ~13% missing data after 8.5% of the patients dropped out just after randomization and 9% during treatment. The drop-out rate is similar to other CBT studies for CFS (22). Missing at random is an assumption not possible to test directly and there was some evidence of higher number of missing responses for the standard CBT due to the larger number of dropouts compared to the other groups. Another limitation was that the intervention groups received different types of CBT regarding both duration and therapeutic content, which made it difficult to make a precise chronologic comparison. The difference in length of therapy also made it difficult to achieve true masking to treatment allocation of the statistician undertaking the analysis of primary outcomes. The intervention groups did not have the same time assessment points after randomization. Hence, measurements and comparisons were not made at the same number of weeks. Given the chronic nature of CFS, a difference of a week or two is unlikely to make a difference to the analyses, but it would have been optimal to compare the groups at the exact same number of weeks throughout the entire trial. Also, due to ethical considerations, we were not allowed to follow the WCL group for more than 18 weeks post baseline before they were offered therapy. Thus, the WLC group was not scored at 1-year follow-up. We considered to measure outcomes at 12 months also for the WLC group but decided not to because they all received 8 weeks of I-CBT immediately after the end of the waiting period, which would contaminate the group. There is limited knowledge of the natural time variation for CFS patients participating in clinical trials, but other studies with long term-follow-up after CBT for CFS show fairly sustained positive effects (20, 64). Both the primary and secondary outcomes were subjective in this study i.e., rated by the participants impressions. The lack of established specific and sensitive biomarkers limits the potentials for valid objective endpoints. Nevertheless, obtained objective tests, such as e.g., indirect VO2 max scores will be presented in later articles.

## Conclusion

The findings of this study suggest that both standard CBT and the shorter I-CBT-intervention, individually delivered, are safe therapies which improve physical function in patients with mild to moderate CFS. Standard CBT also cause positive effects on fatigue. The positive effects sustained for at least 1 year. Thus, patients with mild and moderate chronic fatigue syndrome diagnosed according to the CDC and Canadian Consensus Criteria should be offered individual CBT with a prospect of improved physical function and a safe fatigue symptom relief, even though anxiety and depression symptom scores are normal.

## Data Availability Statement

The raw data supporting the conclusions of this article will be made available by the authors, without undue reservation.

## Ethics Statement

The studies involving human participants were reviewed and approved by The Norwegian regional ethicks commity, NTNU/REK midt, Det medisinske fakultet, Postboks 8905, 7491 Trondheim, NORWAY. REK-project number: 4.2008.2586. The patients/participants provided their written informed consent to participate in this study. Written informed consent was obtained from the individual(s) for the publication of any potentially identifiable images or data included in this article.

## Author Contributions

MG: calculations, figures and tables, writing and reviewing the text, and corresponding author. EF: planning of the project, writing, and reviewing the text. TS and PB: planning of the project and review of the text. JB: statistics, calculations, figures and tables, and review of the text. All authors contributed to the article and approved the submitted version.

## Conflict of Interest

TS was the owner of Coperio, a commercial company, from 2005 to May 2022. The study took place at the Pain Clinic, St Olav's University Hospital, Trondheim, Norway. A number of patients received and diagnosed at St Olav's University Hospital had treatment in offices at Coperio, these patients remained patients of St Olav's University Hospital and no patients admitted primarily to Coperio participated in the study. The Coperio Centre has not delivered individual interpersonal and personality-oriented CBT to patients with CFS/ME or other disorders or syndromes either prior to the study or after the study. The remaining authors declare that the research was conducted in the absence of any commercial or financial relationships that could be construed as a potential conflict of interest.

## Supplementary Material

Supporting Information 1 Study protocol: project description in English.

Supporting Information 2 List of blood test.

Supporting Information 3 Deviations from the study protocol.
