# Corrigendum: Cognitive behavioral therapy improves physical function and fatigue in mild and moderate chronic fatigue syndrome: A consecutive randomized controlled trial of standard and short interventions

**DOI:** 10.3389/fpsyt.2022.1122220

**Published:** 2023-03-13

**Authors:** Merethe Eide Gotaas, Tore C. Stiles, Johan Håkon Bjørngaard, Petter C. Borchgrevink, Egil A. Fors

**Affiliations:** ^1^Department of Circulation and Medical Imaging, Faculty of Medicine and Health Sciences, Norwegian University of Science and Technology, Trondheim, Norway; ^2^National Competence Centre for Complex Symptom Disorders, St. Olav's University Hospital, Trondheim, Norway; ^3^Department of Psychology, Norwegian University of Science and Technology (NTNU), Trondheim, Norway; ^4^Department of Public Health and Nursing, Faculty of Medicine and Health Sciences, Norwegian University of Science and Technology, Trondheim, Norway; ^5^Faculty of Nursing and Health Sciences, Nord University, Levanger, Norway

**Keywords:** CFS, chronic fatigue syndrome, CBT, fatigue, physical function, myalgic encephalitis

In the published article, there was an error in the **Conflict of interest** statement. One of the authors did not disclose commercial links with a company where the study took place. The correct **Conflict of interest** statement appears below.

