# Supplementary material for: Cognitive Behavioral Therapy Improves Physical Function and Fatigue in Mild and Moderate Chronic Fatigue Syndrome: A Consecutive Randomized Controlled Trial of Standard and Short Interventions
Source: Front Psychiatry. 2021 Apr 12;12:580924. doi: 10.3389/fpsyt.2021.580924 (PMC8071989; doi:10.3389/fpsyt.2021.580924)
Supplement: Supporting Information 1 — Study protocol: project description in English. [file Data_Sheet_1.PDF]

Is cognitive behavioural therapy a good treatment for chronic fatigue syndrome?

Summary:

The Norwegian Directorate of Health has requested documentation on treatment for chronic fatigue syndrome, CFS/ME. With this trial, The National Competence Centre for Complex Symptom Disorders (NKSL) wants to contribute to knowledge on whether cognitive behavioural therapy is good treatment for CFS/ME. Even though standard, individual CBT together with graded exercise therapy so far is the best documented treatment, there is no national or international consensus due to few randomised trials, varying quality and moderate evidence. Therefore, our goal is to test the effect of individual standard CBT, in a randomized, controlled 1-year follow-up trial. Our second goal will be to test the effect of a newly developed and shorter individual CBT (I-CBT). If the shorter therapy turns out to be as good as or better than standard CBT, the shorter therapy will be an equal and more cost-effective treatment. Patients with nonspecific fatigue will undergo a thorough, multidisciplinary assessment at the The Department for Pain and Complex Symptom Disorders (ASSL), St. Olav`s Hospital, to be diagnosed with CFS/ME or other fatigue conditions. Patients diagnosed with CFS/ME will be offered treatment in our trial with randomisation to either short I-CBT or standard individual cognitive behavioural therapy or a control group. Primary outcome measures are physical function, health related quality of life and fatigue. NKSL is a research group at ASSL, St. Olav`s Hospital that wants research to be the basis for the operation of its three outpatient clinics; the clinic for pain, the clinic for CFS/ME and the clinic for assessment to work related rehabilitation at Hysnes, Norway.

In our opinion, this study fits perfectly with the announcement:

Clinical research. Transitional research: The health care system needs evidence-based information on treatment for this patient group, that is whether cognitive behavioural therapy is effective. The results can also be transferred to patients with complex symptom disorders. **Interdisciplinarity:** The trial will engage a broad spectrum of professionals both scientifically speaking (the project group) and clinical (multidisciplinary assessment). There are also simultaneous investigative studies of the CFS/ME patients where researchers outside the project group are involved in Regional Ethics Comity (REK) approved projects. ***Randomised controlled trial (RCT):*** The project has a good and clear research method with, among other things, a randomised controlled design, a power calculation that takes into account drop-outs (large patient group) and intention-to-treat (ITT) analysis. ***Biobank:*** The trial involves storing blood samples from all the included participants in a biobank, which provides opportunities for subsequent trials.

Feasibility: The trial has already been going on for two and a half years and inclusion of patients is soon to be completed. The study protocol is registered in Clinicaltrials.gov and has already been referred to as an ongoing trial in a report from Norwegian Research Centre for Health Services. **Relevance for the Health Authority. The use of environment, regional cooperation and interaction:** Patients with fatigue are referred from the entire region of Mid-Norway, and cooperation on these patients is important to ensure good and relevant care. The project can, after completion, contribute to more knowledge on treatment both nationally and internationally. The research group behind the application is an interdisciplinary environment that supports each other and works actively to raise competence in areas that are characterized by weaker research. The research environment represents several institutes at NTNU and cooperates with universities both nationally and internationally. The Norwegian Directorate of Health has in a letter to the Department for Health and Care requested more documentation on treatment for CFS/ME.

Background for this project

Chronic fatigue syndrome (CFS/ME) is characterized by disabling fatigue that exceeds a period of 6 months and which inhibits the individual from daily life's functions without an ongoing strain that can explain the ailments. Additional symptoms include impaired memory, impaired concentration, sore throat, muscle and joint pain, headache, sleep disturbances, and pathologically prolonged symptomatology following stress. The diagnosis criteria for CFS/ME requires that the fatigue must be medically unexplained, newly emerged and with little effect of rest, but usually occurs after physical and mental stress. Some people may be hypersensitive to light, sound and touch, as well as developing irregularities in the autonomic nervous system (1,2). The diagnosis is made based on diagnostic criteria in which specific diagnoses that can cause chronic fatigue should be excluded. The aetiology and pathogenesis of CFS is largely unknown and so far, no consensus has been reached (2,3), but there is a common pinion that the causes of this illness are complex (4).

Epidemiological studies have shown that up to 30% of populations have unspecific, chronic fatigue lasting for more than six months at any given time. According to a report from the Norwegian Research Centre for Health Services (5), there are an estimated 10 to 20,000 inhabitants in Norway who have been diagnosed with chronic fatigue syndrome, and thus a population prevalence of 0.2 to 0.4%. CFS/ME patients frequently see a doctor, averaging 13 consultations per year in the general medical services and 5 consultations per year in secondary care (6). The Norwegian Directorate of Health assumes that fatigue patients have frequent contact with the primary health care in Norway. Approximately half of the patients are expected to get sufficient help in the limits of what can be offered in primary health care, while the rest need additional help (7). Age varies between 40-59 years, and there are more women than men, approximately 3:1 (8,9).

There is no national or international consensus on treatment for chronic fatigue syndrome. Systematic reviews and randomised controlled trials have investigated the effect of several treatments for CFS/ME (10,11,12). CBT and grade exercise therapy (GET) are the only treatments that seems to be favorable (12,13). Even though most of these studies conclude that cognitive behavioral therapy (CBT) increases health related quality of life (HR-QoL) and physical function, fatigue and psychological comorbidity, no treatment has shown high evidence or a curative effect. Objections to CBT for CFS/ME have been targeting decreasing effect at follow up measures, the low number of studies and that evidence is regarded to be “moderate” independent of the outcome measures used (10). A few studies have reported that CBT causes harm or has no effect (14,15). Patient organizations have also been sceptic to CBT and grade exercise therapy, and some self-reported surveys initiated by patient organizations have concluded that CBT is a bad treatment for CFS/ME (16). Thus, they have recommended rest and pacing, but no systematic reviews support this recommendation. There has been a lot of turmoil, especially patients’ organizations, regarding the use of CBT. An evaluation of the treatment effect of CBT can give important information to patients and their next of kin and may correct undocumented patient management. Some patients diagnosed with CFS/ME spend months and years in bead, because of following advice that rest is the way back to normal functioning.

The Department for Pain and Complex Symptom Disorders (ASSL), CFS/ME outpatient clinic, St. Olav`s Hospital, has since 2008 received patients with fatigue for inclusion in a consecutive, randomised, controlled treatment trial with short and long/standard individual cognitive behavioural therapy as intervention. Thus, we will use the collected data from ASSL, and plan to have enough patients included per 01.01.2012 according to conducted power calculation. The study protocol is approved by the Regional Ethics Comity (REK), project number 4.2008.2586, and is also registered in Clinical trials.gov. The trial is also refferd to in a report from the Norwegian Research Centre for Health Services (5). The Norwegian Directorate of Health has referred to this report in a letter to the Department for Health and Care which has requested more documentation on treatment for chronic fatigue syndrome, CFS/ME. Our project has started without research funding as an outpatient offer to this group of patients. The collection of data will soon be completed, and everything is now ready for a PhD candidate to start working on the material.

A short description of the research environment

This project is run by The National Competence Centre for Complex Symptom Disorders (NKSL). Leader of this research group is professor Petter C. Borchgrevink, has gathered competence and resourceful people around the research group which celebrated a ten-year anniversary this year. NKSL, which no subject to St. Olav`s Hospital HF, consists of several part-time employed scientists and professors (see Appendix). The aim of NKSL is to develop guidelines, treatment measures and procedures of cooperation

to avoid that people who are at risk of developing complex symptom disorders develops chronic illness and becomes disabled. The foundation of the research group is built on the biopsychosocial model, with focus on understanding illness and symptoms as a combination of biological, mental and social factors. NKSL has worked for national competence development by arranging classes and seminars for all relevant professions in the health care system. Developing a national Norwegian education for specialist nurses in pain medicine in cooperation with HIST (local nursing school) at the value of 15 student credits is an example of this work by NKSL. This cooperation between NKSL and HIST has now successfully been going on for three years. Scientist connected to NKSL have so far in 2011 and during the entire year of 2010 published 22 and 27 PubMed-registered articles respectively. Meetings to discuss articles and the ongoing work of our PhD-candidates are arranged weekly, and a 2-day long research seminar was recently arranged in the occasion of NKSL`s anniversary. A 2-day gathering for PhD-candidates in February 2012 is also planned. It important to emphasize that NKSL as a National Competence Centre does not receive funding for research, and thus has to apply for funds to be able to start research projects. This project is a part of NKSL`s aim, via clinical close to patient research, to describe, understand and find evidence-based treatment approaches for patients with chronic fatigue syndrome. Experience from handling other complex symptom disorders implies that we also aim for interaction with the primary health service in this project. Our aim is to provide evidence-based advice back to the primary health service within a previously rather weak area of research.

Name and title	*Affiliation*	*Competence/work task*
**Tore Charles Stiles**, Dr.philos, psychologist, professor	Professor at the Institute of psychology, NTNU, member of NKSL, psychologist.	Main supervisor with expertise in cognitive therapy, mental function and mapping tools used in this context. Has developed short CBT, as part of the treatment study. Coauthor
**Petter Christian Borchgrevink**, MD, PhD, Senior consultant, professor.	Senior consultant at The Department for Pain and Complex Symptom Disorders (ASSL), St. Olav`s Hospital<br><br>Supervisor/senior consultant of NKSL, professor at ISB.	Supervisor with expertise in patients with complex symptom disorders, medical conditions. Has participated in the development and design of the study. Coauthor.
**Egil Andreas Fors**, MD, PhD, senior consultant, GP, psychiatrist, scientist.	Senior consultant and psychiatrist at St. Olav`s Hospital, Department of psychiatry.<br><br>Scientist at NKSL, employed at ISM.	Supervisor for the candidate, responsible consultant in relation to the clinical patient offering. Has participated in the development and design of the study. Supervisor to standard CBT. Coauthor.

**Trudie Chalder,**<br><br>professor	Professor in psychology at Kings College i London, UK.	Has developed the Chalder Fatigue Questionnaire scoring tool, made the standard CBT treatment manual at CFS / ME, and participated in the development and design of the study. Professor Chalder will be an international partner for the candidate and has extensive experience from similar studies in the same patient group. Coauthor
**Nils Inge Landrø,** Dr.phil, neuropsychologist and professor	Professor in neuropsychology, University of Oslo. Scientist at NKSL.	Has participated in the design and method development of the study. Has expertise in neuro-psychological function, and co-author in which these studies are part of the project.
**Pål Romundstad,** MD, PhD.	Professor at ISM, NTNU	In charge of method and statistics.

Research questions and hypotheses

Our main research question is whether cognitive behavioural therapy, CBT, is effective treatment for CFS/ME, this can be divided into 2:

1) Does cognitive behavioural therapy improve physical function, fatigue and psychological issues in patients diagnosed with CFS/ME?

2) Is short cognitive behavioural therapy, I-CBT, equal to or better than standard, long CBT intervention in treatment of CFS/ME?

Hypothesis

(1) Standard CBT (CBT) and short CBT (I-CBT) are significantly better compared to the waiting list control group (WLC) in the primary outcome measure physical function in SF-36 and in the secondary outcome measures fatigue and the rest of the SF-36 subdimensions.

(2) There is no difference in effect between I-CBT and standard CBT in the primary outcome measure physical function in SF-36 and in the secondary outcome measures fatigue and the rest of the SF-36 subdimensions.

Secondary research questions

(1) Which baseline factors (beyond the randomised interventions) are associated with physical function and fatigue in the entire CFS/ME selection? (2) Are the intervention effects of CBT for the primary outcome associated with equivalent changes in secondary outcome measures like fatigue, physical fitness, ability to relax, negative affect, pain and number of subjective health complaints? (3) Are the effects of CBT intervention associated with baseline factors like neuropsychological functioning, physical fitness, ability to relax or sociodemographic factors?

Design, methods and analysis

Inclusion and exclusion:

Patients who meet the diagnostic criteria CFS / ME according to the Fukuda CDC 1994 criteria (1) will be offered inclusion in our treatment study and receive outpatient, individual treatment. Exclusion: Absence of fatigue. Age <18 years and> 62 years. Severe somatic or psychiatric disorder. Pregnancy. Problematic drug use. Poor skills in Norwegian language.

Design: A randomised controlled trial is a suitable design to answer our primary research questions on whether CBT has an effect in CFS/ME. The trial is not blinded for intervention, but the investigator is blinded to measurement of the outcome variables. All the groups will be treated equal during the trial, except from exposure to the interventions that are to be evaluated.

Procedure and patient flow:

In this project, patients with nonspecific fatigue referred to The Department for Pain and Complex Symptom Disorders (ASSL) St. Olav`s Hospital, will undergo a thorough interdisciplinary assessment by a physician and a psychologist, physical tests and blood tests to diagnose CFS / ME and categorize other fatigue conditions. Patients who meet the diagnostic criteria for CFS/ME will, after a neuropsychological test, be randomised to one of three groups in the treatment trial following procedures from the Section for Applied Clinical Research (ACR) at NTNU.

Intervention

The intervention consists of short (8 weeks) and standard (16 weeks), cognitive behavioural therapy (CBT) that will be compared to a waiting list control group (WCL).

Figure 1: CFS/ME logistic research project flow chart

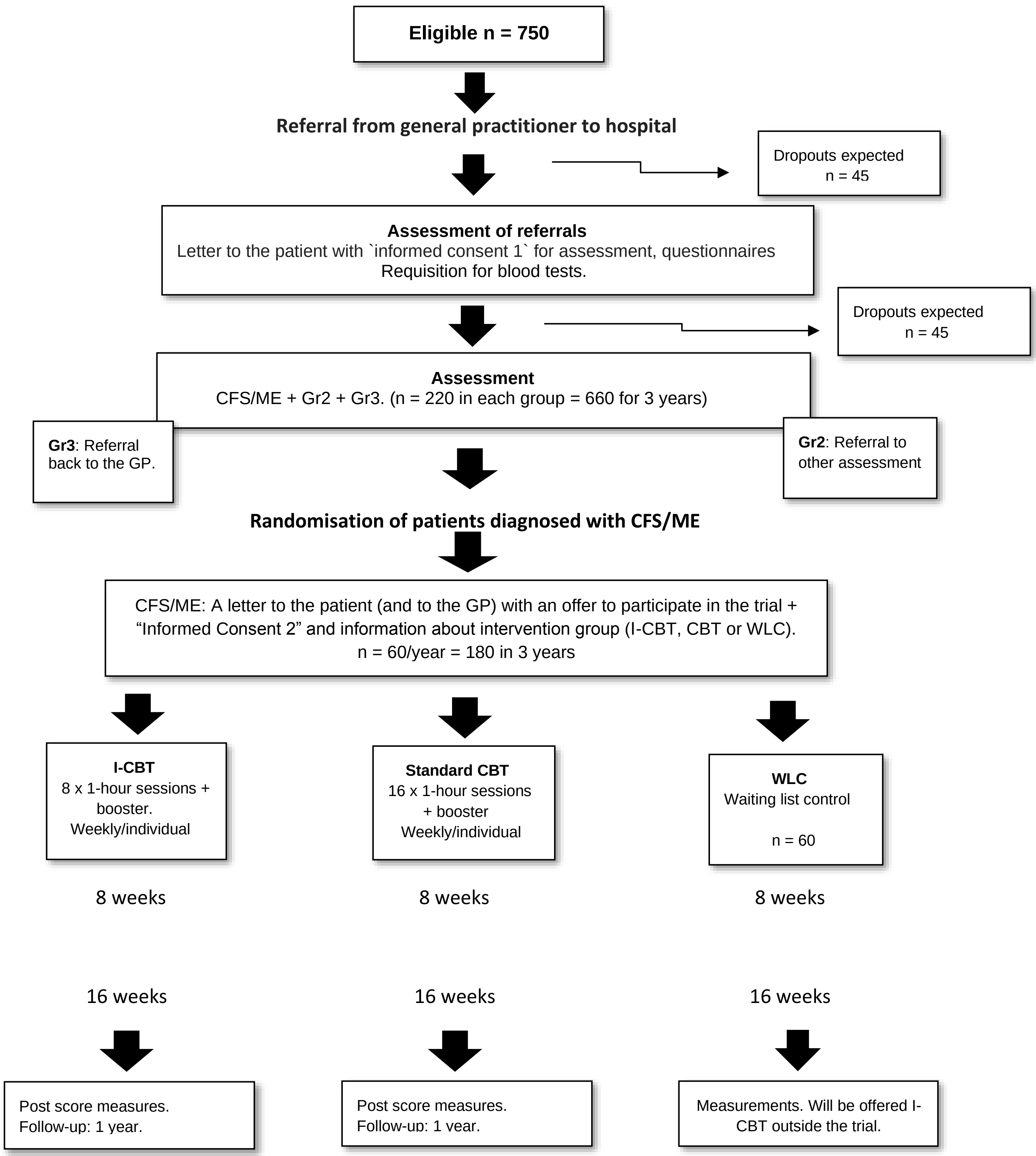

CBT short intervention (I-CBT): The treatment manual is developed by co-author professor Tore Charles Stiles. The intervention consists of eight weekly therapy sessions and a booster session four weeks after the eighth session. The treatment is based on a biopsychosocial model in which elements from interpersonal cognitive therapy and personality-guided therapy are integrated. It assumes that treatment must be individualized to match the prominent personality style of the patient. The patients will be divided into four subtypes: Cautious, achievement oriented, identity or role confused and disease oriented. The overarching goal of the treatment will be to give the patient insight into own personal goals and needs, to know their true inner self, and how to improve their interpersonal relatedness towards others, including family, friends, work relations and health and welfare authorities. The following elements were included in the intervention: (a) information and explanation of the CFS symptoms based on contemporary knowledge on both physical and psychological components with specific emphasis on the goals and needs of the self, its ability to emotion regulate and to actively improve relationships with others, (b) agreement on long-term goals related to self-satisfaction and interpersonal functioning rather than immediate symptom reduction and increase in physical function, and (c) focus on individualized personality-guided and interpersonally oriented factors that are assumed to create painful and difficult experiences to the individual. The patient will, in home assignments, test out various types of cognitive techniques and perform various behavioral experiments that are well known in the field of cognitive therapy. During the last two treatment sessions, one will focus on how one can continue what one has learned in the therapy and how to prevent relapse. Supervision in I-CBT will be given by Professor Tore Stiles once a month in groups, as well as individually when needed. All treatment sessions will be taped for quality control and guidance.

Standardised CBT, long intervention: The intervention consists of 16 weekly sessions plus a booster session four weeks after the 16th session, ad modum "Chalder/Kings College". The actual therapy manual is based on previous manuals from similar trials and associated with the fear avoidance theory (FA) of CFS. The FA theory focuses on the link between cognitive and behavioural responses. The following main elements are included in the intervention: (a) information and explanation of the CFS symptoms based on contemporary knowledge on both physical and psychological components, (b) agreement on goals for the

treatment (c) reading of self-help guides like *Coping with Chronic Fatigue* (d) avoidance of excessive activity or rest and/or sudden change in activity, (e) planning of regular, predictable, continuous activity to prohibit deconditioning, (f) recovering self-confidence and self-control by starting with a five- minute walk morning and night followed by gradual development (GET), and (g) classical CBT-procedures. A treatment manual for SCBT has been developed following Professor Trudie Chalders guidance. in use at the Division of CFS/ME, Kings College, UK (17). There will be regular supervision of the therapists in relation to this manual. All treatment sessions will be taped which will allow for quality control and satisfactory CBT guidance. Appropriate use of standard CBT will be quality assured using independent observers with CTACS quality assurance instrument afterwards (18) so that all therapists are quality assured as CBT therapists. Guidance is provided by a specialist in CBT guidance (psychiatrist, Dr. Egil Fors) regularly once a month in a group, as well as individually when needed.

Control group (WLC): The control group is a waiting list control group in which the participants will be asked to wait for 16 weeks after primary assessment and randomisation before they are post-scored and then offered eight weeks of I-CBT outside the study. The ideal would have been a control period up to one year without intervention, but this will not possible due to ethical considerations.

Assessment tools/outcome measures

The patients will be asked throughout the project to fill out questionnaires about their health and overall condition. The survey focuses on both mental, physical and social conditions.

Primary outcome measure

The primary outcome measure of this trial will be one of eight subdimensions, physical function, measured by "SF-36" which is a 36-item questionnaire that produces scales for both mental and physical health (19,20). The questionnaire has been used previously and is well validated in similar studies (21), is sensitive to treatment change (22) and is a sufficient measure of functional status (23).

Other outcome measures will be "Fatigue" measured by Chalder Fatigue Scale (score 0-3 with 11 items, max score/fatigue = 33 points) (24) and mental function in SF36 (0-100) (20). Outcome success criteria will be > 10 points improvement in physical function in SF36 analyzed by a 0-100 scale, where 100 is best and 0 is worst (13), 50% recovery or a score of 75 points (25).

Figure 2: Examples of outcome for physical function and fatigue (n = 3):

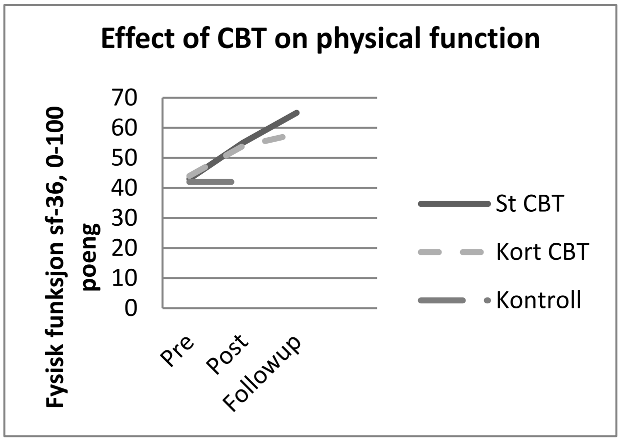

For both figures from top to bottom: Standard CBT, Short I-CBT and Control group (WLC).

Effect of CBT on fatigue

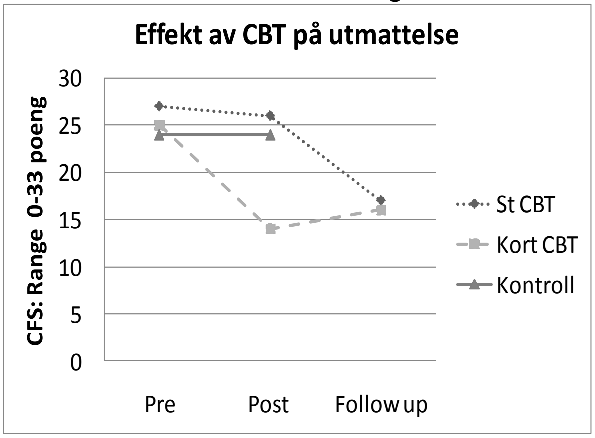

Secondary outcomes:

Physical condition VO2 max (26), pain (NRS), tender points, negative affect (HADS), an “amount” of subjective health complaints (SCL-90, SHC) (27,28). We will be able to combine these above-mentioned outcomes, for example, changes in "fatigue" with changes in specific cognitions and behaviour responses as moderators. In addition, there will be baseline targets, including neuropsychological parameters, relaxation capacity (GFM-52) (29) and psychometric measures as predictors of the main outcome measurements.

Analyses:

Calculation of sample size and power: We have calculated sample size assuming a mean change in SF-36 score of 10, 5, and 0 from baseline of respectively short I-CBT, standard CBT, and WLC group, a \`within cell\` standard deviation (SD) on 15 and a correlation between baseline and post score on 0.3 (r-squared on 0.1). For a two-sided test with 5 % significance level and 80 % power, we calculated that we needed 40 patients in each group, 120 in total. Assuming a maximum drop-out level of 25 %, we calculated that we needed an additional number of 18 patients in each group, 174 patients in total. A mean change in SF-36 score of 10, 10, and 0 for short, standard, and WLC groups would increase power to 90 %.

Statistical analyses:

ANCOVA and "mixed random effects regression" will be used to compare the post-score / outcome variables in the three intervention cohorts (among others physical function in SF36). All analyzes will be adjusted for baseline scores with postscore as outcome variables. The three intervention cohorts will be compared at X, Y and Z months. We will perform separate analyzes for each follow-up time (ANCOVA) as well as combined analyzes for all times (mixed random effects regression). The results will be presented as "least-squares means with standard errors (SE). Differences between cohorts will be reported with 95% confidence interval (CI).

Intention to treat:

All patients will be accounted for at the end of the trial, i.e. according to “Intention to treat” (ITT) analysis.

Schedule and timeline for dissemination of the research results:

In addition to publishing articles in international scientific journals, the project will also aim to generate informative articles, for example, articles in Norwegian journals that can be used in the primary health care. In addition, we have established a collaboration with the Norwegian Electronic Medicines Manual (NEL), and thus our research will eventually also benefit primary health service and its patients in the search for understanding of symptoms and any investigation or treatment strategy. A PhD candidate will be able to attend national and international meetings and conferences with poster presentations and participate in workshops, for example in the Society of Behavioral Medicine (SBM) and at the International Conference of CFS / ME, which will be held in 2013.

Schedule: The aim is to start processing data from the trial on 01.01.2012. The project will last for another 3 years, to 01.01.2015. *Publishing*: The CFS / ME project is planned to generate 3 articles in total, see below:

	**Spring 2012**	**Fall 2012**	**Spring 2013**	**Fall 2013**	**Spring 2014**	**Fall 2014**
**Data collection**	**X**	**X**				
**Data processing**	**X**	**X**	**X**	**X**		
**Data analysis**		**X**	**X**	**X**	**X**	**X**
**Publications**		**X**	**X**	**X**	**X**	**X**

Paper 1: Short versus standard individual cognitive behaviour therapy for chronic fatigue syndrome: a consecutive, randomised controlled trial. *Primary outcomes are Global quality of life (SF-36), physical and, mental functioning SF-36) and fatigue (Chalder Fatigue Scale). Secondary outcomes are mood (HADS), pain (NRS) and physical condition (VO2 max).*

Paper 2: Predictors of treatment outcome after short and standard individual cognitive behaviour therapy for patients with chronic fatigue syndrome in tertiary care: a consecutive, randomised controlled trial. Predictors: (besides the intervention): Demographics, neuropsychology, pain (NRS) and tender points, mood/psychometrics, VO2 max, tension/flexibility (GFM-52) and number of health complaints (SCL-90/SHC).

Paper 3: Does cognitive behaviour therapy improve physical condition and reduce fatigue in patients with chronic fatigue syndrome? The role of physical activity. If CBT improves Fatigue, is this effect mediated by improvement in VO2 max or mood? Dependent variable: Fatigue (Chalder Fatigue Scale), physical condition (indirectly Åstrand`s test), depression (HADS).

References:

(1) Fukuda K, Strauss SE, Hickie I et al. The chronic fatigue syndrome, a comprehensive approach to its definition and study. Ann Intern Med 1994; 121: 9 53 – 9. **(2)** Carruthers BM, van de Sande MI, De Meirleir KL et al. Myalgic encephalomyelitis: International Consensus Criteria. J Intern Med. 2011 Jul 20. doi: 10.1111/j.1365-2796.2011. (**3)** Afari N, Buchwald D. Chronic fatigue syndrome: a review. American Journal of Psychiatry 2003;160;2:221–36. **(4)** Wyller VB, Bjørneklett A, Brubakk Q, Festvåg L, Follestad I, Malt U, Malterud K, Nyland H, Rambøl H, Stubhaug B, Larun L: Diagnostisering og behandling av kronisk utmattelsessyndrom/ myalgisk encefalopati (CFS/ME). Rapport fra Kunnskapssenteret nr 09 - 2006. **(5)** Larun L, Brurberg KG, Fønhus MS, Kirkehei I. Behandling av kronisk utmattelsessyndrom CFS/ME. Notat 2011. ISBN 978-82-8121-416-3. **(6)** Lloyd A, Pender H: The economic impact of chronic fatigue syndrome. Med J Australia 1992, 157:599-601.**(7)** Sosial- og Helsedirektoratet 2007: http://www.helsedirektoratet.no/ fagnytt/ nasjonalt _ kompetansenettver_for_pasienter_med_kronisk_utmattelsessyndrom_foresl_s_70629. **(8)** Prins JB, van der Meer JW, Bleijenberg G. Chronic fatigue syndrome. Lancet. 2006 Jan 28;367(9507):346-55. Review. **(9)** Dinos S, Khoshaba B, Ashby D, White PD, Nazroo J, Wessely S, Bhui KS. A systematic review of chronic fatigue, its syndromes and ethnicity: prevalence, severity, co-morbidity and coping. Int J Epidemiol. 2009 Dec;38(6):1554-70. **(10)** Reid S, Chalder T, Cleare A, Hotopf M, Wessely S. Extracts from “Clinical Evidence”: chronic fatigue syndrome. BMJ 2000; 320:292–96. (**11)** Whiting P, Bagnall A, Sowden A, Cornell J, Mulrow C, Ramirez G. Interventions for the treatment and management of chronic fatigue syndrome: a systematic review. JAMA 2001; 286: 1360–68. (**12)** Price JR, Mitchell E, Tidy E, Hunot V. *Cognitive behaviour therapy for chronic fatigue syndrome in adults.* Cochrane Database Syst Rev. 2008 Jul 16;(3):CD001027. **(13)** O'Dowd H, Gladwell P, Rogers CA, Hollinghurst S, Gregory A. Cognitive behavioural therapy in chronic fatigue syndrome: a randomised controlled trial of an outpatient group programme. Health Technol Assess. 2006 Oct;10(37):iii-iv, ix-x, 1-121. **(14)** Twisk FN, Maes M. A review on cognitive behavorial therapy (CBT) and graded exercise therapy (GET) in myalgic encephalomyelitis (ME) / chronic fatigue syndrome (CFS): CBT/GET is not only ineffective and not evidence-based, but also potentially harmful for many patients with ME/CFS. Neuro Endocrinol Lett. 2009;30(3):284-99.Review. **(15)** Núñez M, Fernández-Solà J, Nuñez E, Fernández-Huerta JM, Godás-Sieso T, Gomez-Gil E. Health-related quality of life in patients with chronic fatigue syndrome: group cognitive behavioural therapy and graded exercise versus usual treatment. A randomised controlled trial with 1 year of follow-up. Clin Rheumatol. 2011 Mar;30(3):381-9.

(16) Action for M.E.: Severely neglected ME in the UK. London, Action for M.E.; 2001. **(17)** Kings College CFS/ME senter, London: website: kcl.ac.uk/cfs. **(18)** Barber, J. P., Liese, B. S., & Abrams, M. J. (2003). Development of the cognitive therapy adherence and competence scale. Psychotherapy Research, 13, 205-221. **(19)** Ware JE, Sherbourne CD (1992) The MOS 36-item short-form health survey (SF-36). I.

Conceptual framework and item selection. Med Care 30:473–483. **(20)** McHorney CA, Ware JE Jr, Raczek AE. The MOS 36-item short-form health survey (SF-36): II. Psychometric and clinical tests of validity in measuring physical and mental health constructs. Med Care 1993;31:247–63. **(21)** Deale A, Chalder T, Marks I, Wessely S. Cognitive behaviour therapy for chronic fatigue syndrome: a randomised controlled trial. Am J Psychiatry 1997;154:408–14. **(22)** Reeves WC, Lloyd A, Vernon S, Klimas N, Jason LA, Bleijenberg G, et al. Identification of ambiguities in the 1994 chronic fatigue syndrome research case definition and recommendations for resolution. BMC Health Serv Res 2003;3:25. **(23)** Buchwald D, Pearlman T, Umali J, Schmaling K, Katon W. Functional status in patients with chronic fatigue syndrome, other fatiguing illnesses, and healthy individuals. Am J Med 1996;101:364–70. **(24)** Chalder T, Berelowitz G, Pawlikowska T, et al. Development of a fatigue scale. J Psychom Res. 1993;37:147-153. **(25)** White PD. What causes chronic fatigue syndrome? BMJ 2004; 329: 928-9. **(26)** Wiborg JF, Knoop H, Stulemeijer M, Prins JB, Bleijenberg G. How does cognitive behaviour therapy reduce fatigue in patients with chronic fatigue syndrome? The role of physical activity. Psychol Med. 2010 Jan 5:1-7. **(27)** Knoop H, Prins JB, Moss-Morris R, Bleijenberg G. The central role of cognitive processes in the perpetuation of chronic fatigue syndrome. J Psychosom Res. 2010 May;68(5):489-94. Epub 2010 Mar 16. **(28)** Fredheim OM, Borchgrevink PC, Landmark T, Schjødt B, Breivik H. A new schedule for the inventory of pain. Tidsskr Nor Laegeforen. 2008 Sep 25;128(18):2082-4. **(29)** Kvåle A. Global Fysioterapi Metode (GFM-52) og Den Omfattende Kroppsundersøkelsen (DOK) - likheter og ulikheter. Bokkapittel i Festskrift til Berit H. Bunkan. Utgitt av Høyskolen I Oslo 2009.

* http://niceguidelines.blogspot.com/2011/01/new-randomised-controlled-trial-shows.html
