# Supplementary material for: Cognitive Behavioral Therapy Improves Physical Function and Fatigue in Mild and Moderate Chronic Fatigue Syndrome: A Consecutive Randomized Controlled Trial of Standard and Short Interventions
Source: Front Psychiatry. 2021 Apr 12;12:580924. doi: 10.3389/fpsyt.2021.580924 (PMC8071989; doi:10.3389/fpsyt.2021.580924)
Supplement: Supporting Information 2 — List of blood test. [file Data_Sheet_2.PDF]

Full list of blood tests

Clinical-chemical blood tests:

Hemoglobin, erythrocyte sedimentation rate, white blood cells differential count, blood platelets, iron, transferrin, transferrin saturation and transferrin receptor, ferritin, Na, K, Ca, P, Mg, blood glucose, albumin, CRP, ALAT, ASAT, GT, Bilirubin ALP, LD, creatinine, CK, vitamin B12, folate, Vit. D, T4, TSH, cortisol.

Immunological test:

Immunoglobulins, IgG, IgM, IgA, total IgE, ANA-screening, rheumatoid factor, anti-transglutaminase anti body.

Microbiological tests:

Serology: EBV (Epstein-Barr virus), CMV(Cytomegalovirus), VZV (Varicella Zoster virus), HSV (Herpes simplex virus), HIV, Toxoplasma, Borrelia, Mycoplasma pneumonia*, Chlamydophila pneumonia*, Hepatitis B and C, Parvovirus B19, PCR (in case of positive serology): Human herpesvirus 6, EBV, CMV, parvovirus B19.
