# Supplementary material for: Cognitive Behavioral Therapy Improves Physical Function and Fatigue in Mild and Moderate Chronic Fatigue Syndrome: A Consecutive Randomized Controlled Trial of Standard and Short Interventions
Source: Front Psychiatry. 2021 Apr 12;12:580924. doi: 10.3389/fpsyt.2021.580924 (PMC8071989; doi:10.3389/fpsyt.2021.580924)
Supplement: Supporting Information 3 — Deviations from the study protocol. [file Table_1.DOCX]

**Deviations from the trial study protocol.**

Changes in outcome measures

1. According to the 36-Item Short Form Health Survey questionnaire developers, a global measure of health-related quality of life such as the “SF-36 Total/Global/Overall Score/HR-QoL” cannot be generated from the questionnaire (1). Thus, we do not report “health-related quality of life/HR-QoL” as an outcome as written in the study protocol.

Change in members of the project group

2. There was a change of staff during the project period. Professor Pål Romundstad was responsible for methods and statistics in the project as written in the study protocol, but professor Johan Håkon Bjørngaard performed the final analyses of outcome data due to change of staff at NTNU.

Professor Nils Inge Landrø has not participated in the work with this article but has been a part of the project group as described in the study protocol.

Professor Trudie Chalder has not participated in the work with this article but has been a part of the project group as described in the study protocol.

Change in schedule and plan for dissemination of research results

3. Publication of results have been delayed due to non-academic reasons.

Changes in content of the three planned articles

4. The project generated a lot of data. In this article (paper 3), we have answered our main research question whether CBT can improve physical function and fatigue in CFS/ME.

The second part of the planned article 3 “*If CBT improves Fatigue, is this effect mediated by improvement in VO2 max or mood? Dependent variable: Fatigue (Chalder Fatigue Scale), physical condition (indirect Åstrand`s test), depression (HADS)”,* will be explored and answered in a separate article due to considerations regarding the amount of data presented in one article.

5. Addition to planned statistical analyses

Due to repeated measures of outcomes, we also used a multilevel linear regression model with random slopes in STATA 11 for Windows (Stata Corp., College Station, TX). This method uses all available information during follow-up and is less susceptible to bias from missing responses under the assumption of missing at random.

**References**

1. Lins L, Carvalho FM. SF-36 total score as a single measure of health-related quality of life: Scoping review. *SAGE Open Med*. 2016; 4:2050312116671725. Published 2016 Oct 4. doi:10.1177/2050312116671725
